# Supplementary material for: Naphthyridine Derivatives Induce Programmed Cell Death in Naegleria fowleri
Source: Pharmaceuticals (Basel). 2021 Oct 1;14(10):1013. doi: 10.3390/ph14101013 (PMC8540127; doi:10.3390/ph14101013)
Supplement: Supplementary file 1 [file pharmaceuticals-14-01013-s001.zip › pharmaceuticals-1390679-supplementary.pdf]

Supporting Information

Table of contents

Contents	Page
**Figure S1.** *N. fowleri* (ATCC30808 and ATCC30215) trophozoites incubated with the IC_{50} of Compound 3 and Negative control	S1
**Figure S2.** Higher magnification of Fig. 4	S2
**Figure S3**. Higher magnification of Fig. 5	S3
**Figure S4**. Higher magnification of Fig. 6	S4
**Figure S5**. Higher magnification of Fig. 7	S5

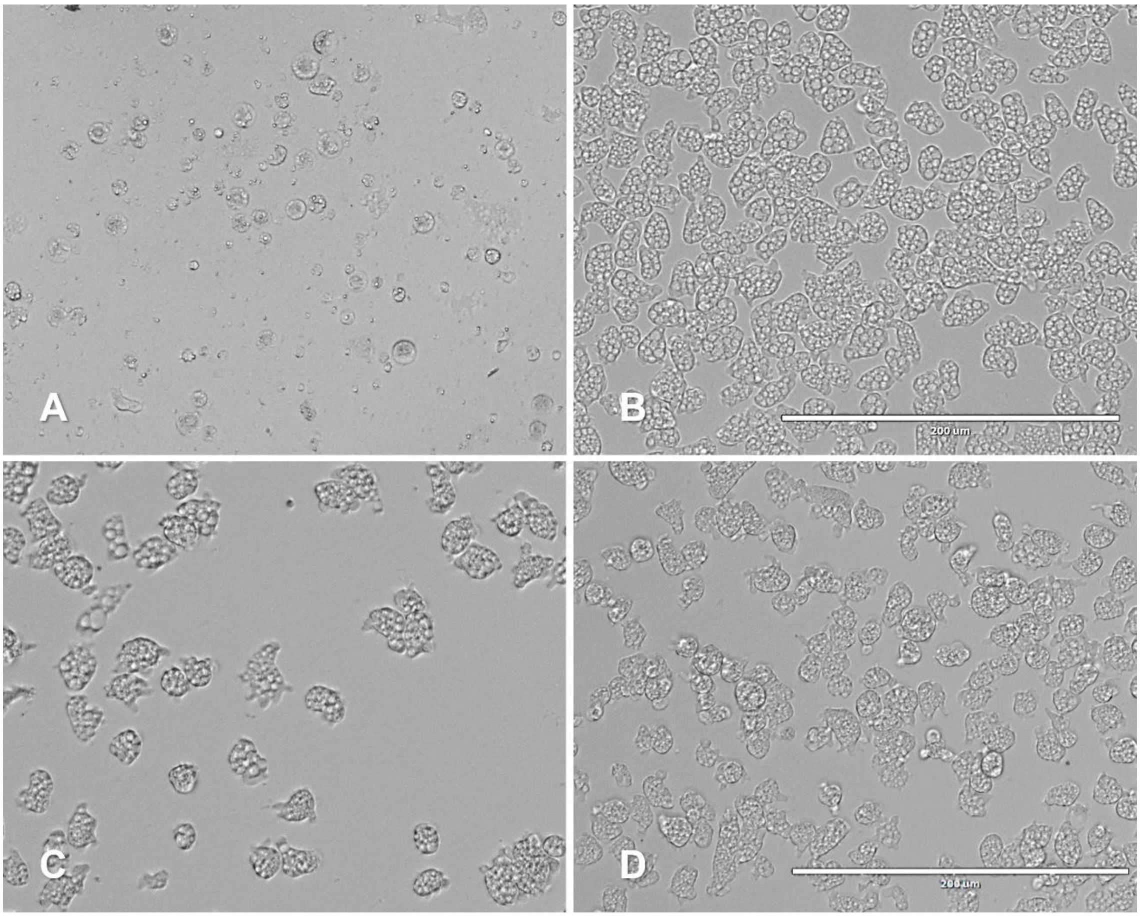

Figure S1. Growth inhibition of *N. fowleri* (ATCC® 30808™) trophozoites at 48 hours at IC_{50} concentration. compound **3** (**A**), negative control (**B**) and *N. fowleri* (ATCC® 30215™) trophozoites at 48 hours at IC_{50} concentration. compound **3** (**C**), negative control (**D**) respectively. All images (**x20**) are representative of the population of treated amoeba and are based on the live cell imaging microscope EVOS FL Cell Imaging System AMF4300, Life Technologies, Spain. Scale bar represent 75 µm

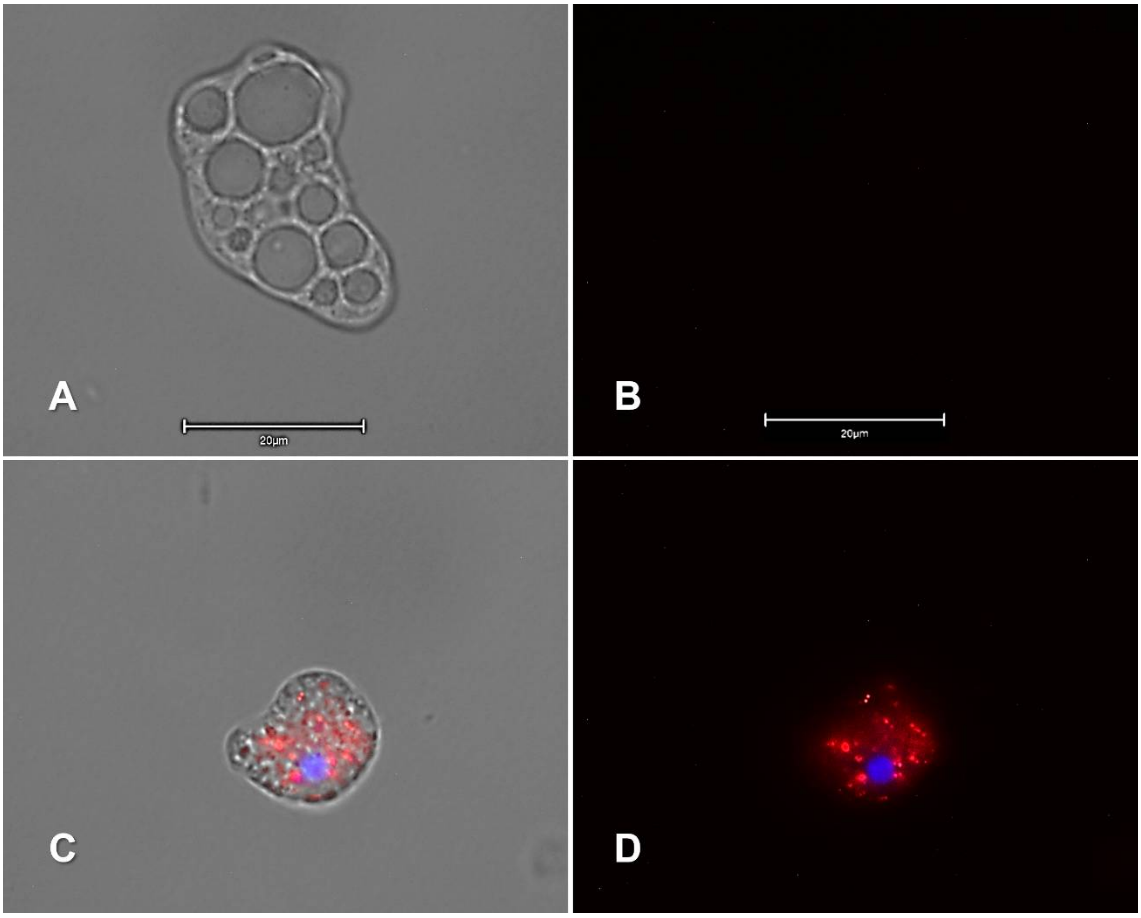

Figure S2. Higher magnification of Fig. 4. Images (x**100**) showing apoptotic cells with high blue and red fluorescence. Overlay channel with visible (A,C), overlay with DAPI and RFP channels (B,D). Images are representative of the cell population observed in the performed experiments. Images were obtained using an EVOS FL Cell Imaging System AMF4300, Life Technologies, Spain.

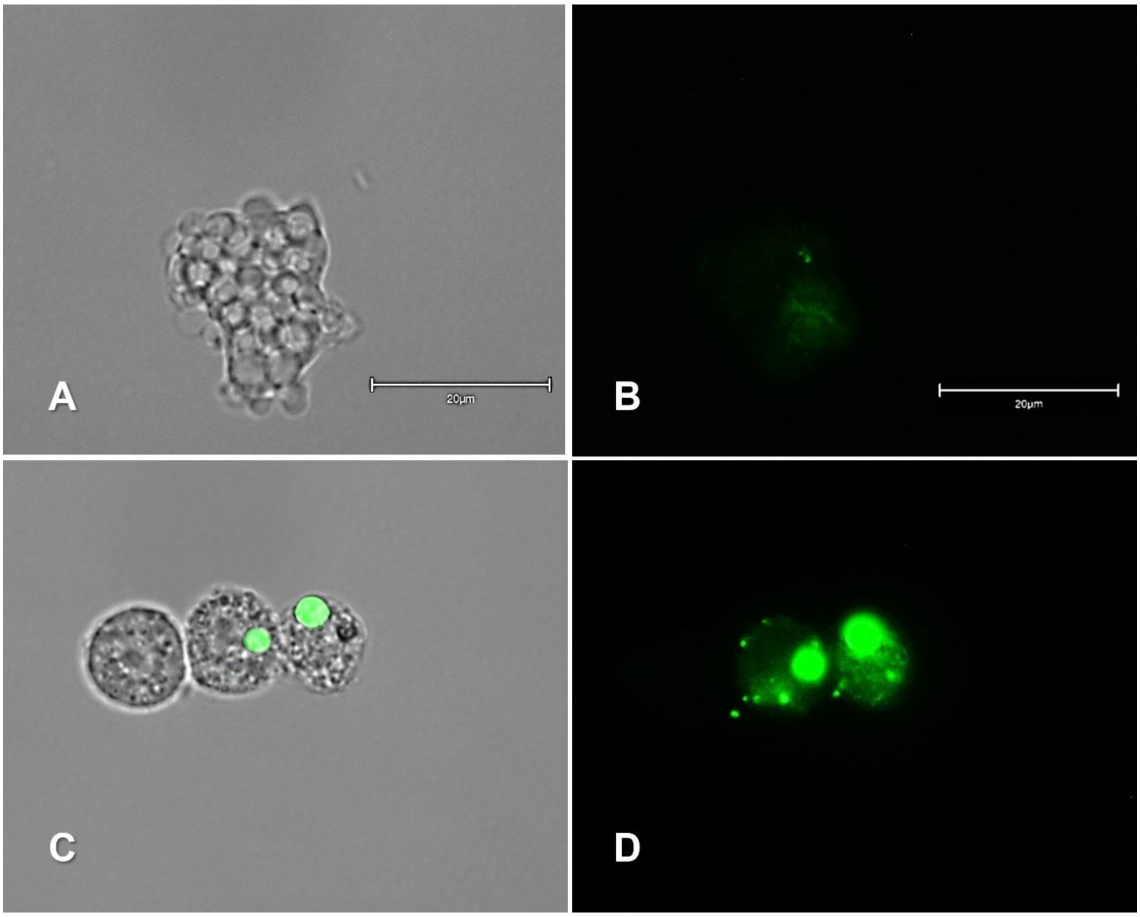

Figure S3. Higher magnification of Fig. 6 (x**100**). Overlay with visible (A,C) and without visible channel (B,D). Images are representative of the cell population observed in the performed experiments. Images were obtained using an EVOS FL Cell Imaging System AMF4300, Life Technologies, Spain.

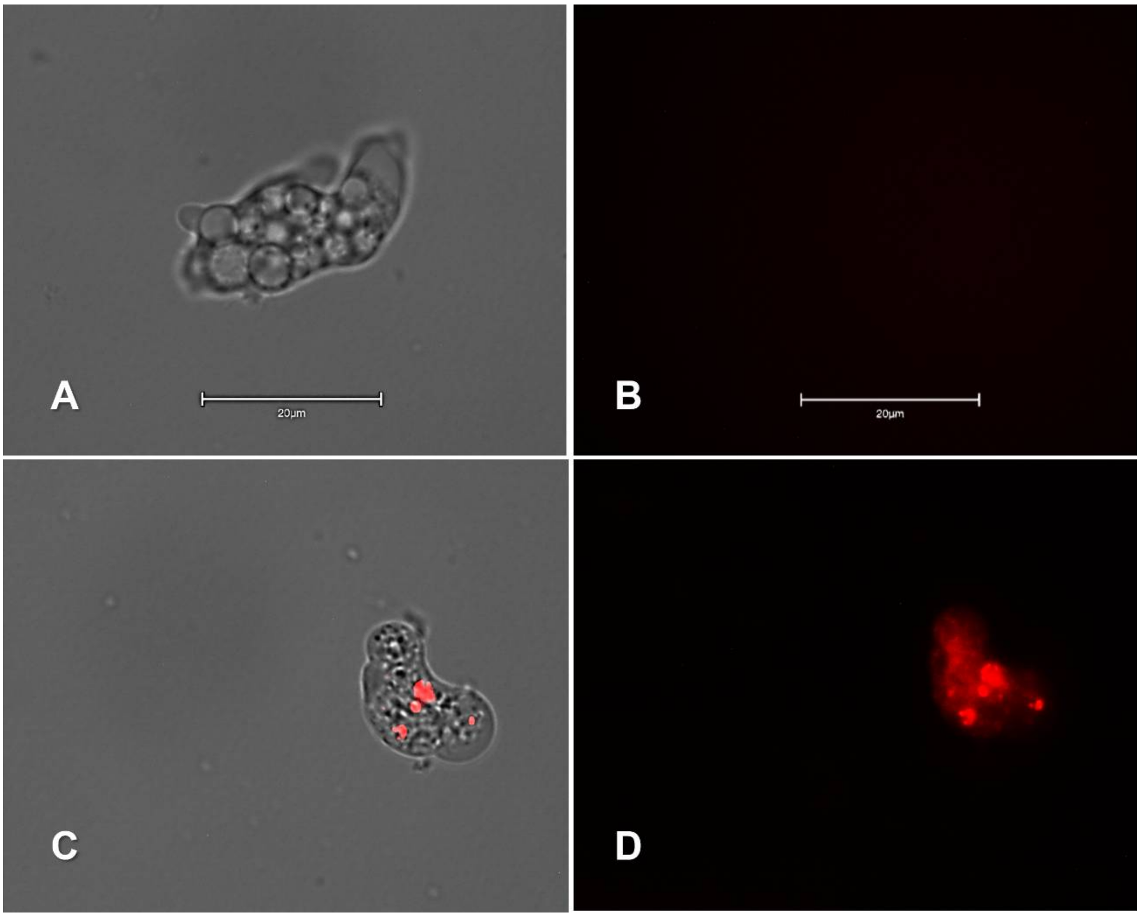

Figure S4. Higher magnification of Fig. 6 (x**100**). Overlay with visible (A,C) and without visible channel (B,D). Images are representative of the cell population observed in the performed experiments. Images were obtained using an EVOS FL Cell Imaging System AMF4300, Life Technologies, Spain.

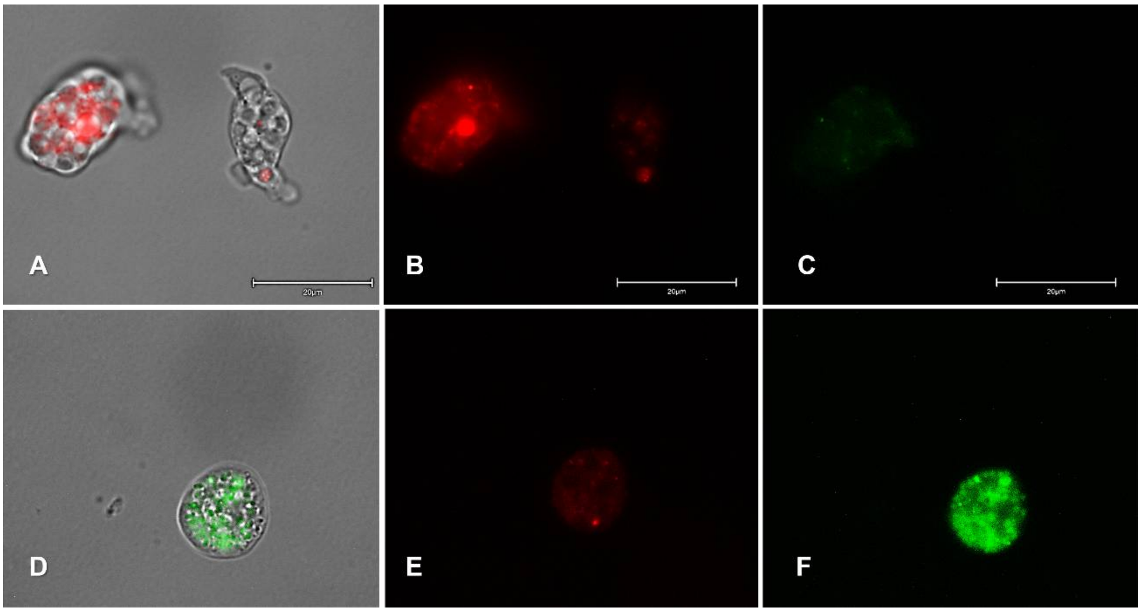

Figure S5. Higher magnification of Fig. 7 (X**100**). Overlay with visible (A,D). Red Channel (B,E) JC-1 dye aggregated form in the mitochondria. Green Channel (C,F) JC-1 dye monomeric form in the cytoplasm. Images are representative of the cell population observed in the performed experiments. Images were obtained using an EVOS FL Cell Imaging System AMF4300, Life Technologies, Spain.
